# Case of a Remarkably Large Tumour Found in the Cavity of the Abdomen

**Published:** 1786

**Authors:** Joshua Fisher

**Affiliations:** *From the* Memoirs of the American Academy of Arts and Sciences


VIII. *Cafe of a remarkably large Tumour found in the Cavity of the Abdomen. By* Jofhua Fifher, *F. A. A. and M. S.* — From the *Memoirs of the American Academy of Arts and Sciences*, Vol. I. 4to. Bofton, 1785.

THE fubject of the following memoir was a woman, of ftrong habit, rather fpare than grofs, and of an active difpofition. She lived in a married ftate from early life, but never had a child; was not peculiarly fubject to any diforder, except fome florid cutaneous eruptions, till at about the ufual time of life the the catamenia ceafed. Soon after that period fhe became fenfible of an unufual fulnefs in the abdomen, which continued almoft imperceptibly increafing, without any difagreeable fymptoms, till fhe was near fixty years old, about two years before her death. She then complained of a pain in the left hypochondriac region, which became fenfibly tumefied; the pain and diftenfion from thence increafed, and fpread over the whole abdomen.

I firft faw her between four and five months before her death; I found the abdomen very large and tenfe, efpecially on the left fide, which was the moft painful: the veffels of her head and arms were full and turgid, while an inanition had taken place in the lower extremities, with a variety of fymptoms arifing from an unequal diftribution of the circulating fluids and nervous influence. But the immediate caufe of her principal complaints was an inflammation, which appeared to be feated before the rectum; although fcarcely any part of the abdomen was exempt from attacks of the moft excruciating pain at intervals, yet the lower and pofterior parts were principally affected; and while a fuppuration was forming, (which had probably taken place feveral times before before I ſaw her) the pain darted a little backward and downward, and terminated in the rectum, from which a quantity of bloody pus was, at length, diſcharged. Her bowels were generally conſtipated, and when the ſymptoms were the moſt ſevere, the diſcharge of urine was ſmall, and attended with pain.

The principal medicines that I made uſe of were, the ſaline draught, and *Ol. Ricin.* with anodynes occaſionally. In the ſpace of a few weeks the ſymptoms of inflammation diſappeared, the ſecretions and excretions became free, and, by the uſe of the bark and gentle exerciſe, ſhe recovered a conſiderable degree of ſtrength, the ſymptoms ariſing merely from diſtention and compreſſion being very tolerable. This truce laſted about two months: a ſudden ſuppreſſion of perſpiration was followed by a return of her complaints with redoubled violence, and ſome paralytic ſymptoms. On the twelfth day of her relapſe ſhe diſcharged a conſiderable quantity of purulent matter, intermixed with ſome blood and fæces, but without any relief, and ſhe died on the fourteenth. In her laſt ſtruggles, which were very violent, ſhe diſcharged (probably *per vaginam*) a quantity of darkiſh water, reſembling high-coloured urine,

 amountamounting, by eftimation, to at leaft two gallons. This difcharge confiderably reduced the bulk of the abdomen, efpecially of the left fide.

The day after her death I went about noon, with the Rev. Mr. Cutler, of Ipfwich, and Dr. Spafford, of Beverly, to open the body. We expected that it would not be buried till the day following; but, to our great difappointment, found it was to be interred the fame afternoon: at length, however, we were allowed an hour to examine it.

On opening the abdomen, which now appeared about as large as in the laft month of pregnancy, a preternatural fubftance prefented itfelf, fituate a little more to the right fide than to the left, and occupying nearly the whole cavity. After feparating it from the contiguous parts, to all of which it firmly adhered, its general figure appeared to be that of a cone or pear; the lower part (fuppofing the body erect) being within the pelvis, was very nearly conical; the upper part was divided by two grooves, running in a right line from its bafis, towards the apex, the one on its anterior, the other on its pofterior fide, dividing it into two lateral portions, and giving it the appearance of its having been formed by two globular bodies bodies compreffed together. The pofterior groove correfponded to the projection of the fpine, to which it adhered ; the anterior groove was wider, and the protuberances on each fide fomewhat larger. The bafis lay above the kidneys, and the apex nearly low enough to form a tumour *in perinæo*. By cutting into it, it appeared to be an uniform fchirrus, of a cineritious colour, with veffels, or rather perforations, interfperfed, for conveying the circulating fluids. From its fituation, connexions, and figure, it appeared to have originated in both the ovaria; the two fchirrofities in procefs of time having united, and, meeting with the leaft refiftance from below, extended into the pelvis till the cavity was completely filled. It weighed upwards of ten pounds and a quarter, averdupois weight. We made fome obfervations on the neighbouring vifcera; but, for want of time, they were unavoidably very imperfect.

The vefica urinaria was moderately diftended with urine, forming a tumour over the offa pubis, the neck being compreffed between the pubis and the fchirrus. Its whole anterior furface was firmly attached to the peritoneum, and the pofterior to the fchirrus, except a fmall portion in the middle of it, which adhered to the uterus, where that viſcus intervened. The ureters, for the ſpace of two or three inches, ſeemed to lie looſe, and contained a ſmall quantity of urine; their coats being thin and conſiderably diſtended. They then entered upon the ſchirrus, one on each ſide, involved in a membrane hereafter mentioned, running obliquely forward and downward till they entered the bladder. After they were connected to the ſchirrus, their appearance was the reverſe of the former: to enable them to take the circuit, they had been greatly extended, and their diameters were exceedingly ſmall. The uterus was extended in a right line upon the ſchirrus from its baſis (to which the fundus uteri adhered) through the anterior groove, almoſt to its apex, continuing about an inch and a quarter wide, and firmly attached through its whole length. Its coats appeared in a natural ſtate, except that they were thinner by reaſon of their extenſion.

The appearance of the tubæ fallopianæ was ſo unnatural, that they were ſcarcely diſtinguiſhable. We found a membranous covering cloſely embracing the baſis, the anterior, and lateral ſides of the ſchirrus, extending downward nearly to the pelvis; and by its upper and lateral edges adhering to the ſchirrus, it ſeemed to ariſe from the uterus, which was ſituated in the middle of it, and its external membrane was a continuation of the external membrane of the uterus. Through this expanſion on the baſis of the ſchirrus were ſeveral ſmooth, but irregular, apertures, of about two inches circumference, through which the baſis of the ſchirrus appeared; it was here about a quarter of an inch thick, its fibres fleſhy and variouſly convolved; the lower part of it was much thinner, and purely membranous. That part of this tegument which was ſpread upon and near the baſis appeared to be formed by a diſtention of the fallopian tubes included in the peritoneum, or common membrane of the abdomen, and the remainder of it by an expanſion of thoſe duplicatures of the ſame membrane, uſually termed ligamenta lata.

On the poſterior ſide the ſchirrus was attached to the aorta, vena cava, &c.; to the rectum, which was protruded to the right ſide of the ſpine, it adhered for the ſpace of ſeven or eight inches: near the baſis the adheſion was ſtrong; but a little lower the connecting membrane contained a quantity of grumous blood, appeared appeared putrid, and in ſome places was deſtroyed.

We wiſhed to have aſcertained the ſource of the water which the patient diſcharged at the time of her death: by the appearance of the viſcera there had been none diffuſed in the cavity of the abdomen; and, from the circumſtances above mentioned, it muſt have been contained in the left hypochondrium. In removing the ſchirrus we found a membrane (a ſac to appearance) adhering to it, which we cut through, and ſuppoſed to be an empty hydropic cyſt; but the ſchirrus at that inſtant engroſſed our attention, and people ſoon collecting to attend the funeral, all farther examination was prevented.

We obſerved nothing extraordinary in any of the other viſcera: the omentum, as is uſual in tabid caſes, was nearly waſted, and a number of the meſenteric glands were enlarged and indurated.

